# Effect of an Enhanced Self-Care Protocol on Lymphedema Status among People Affected by Moderate to Severe Lower-Limb Lymphedema in Bangladesh, a Cluster Randomized Controlled Trial

**DOI:** 10.3390/jcm9082444

**Published:** 2020-07-30

**Authors:** Janet Douglass, Hayley Mableson, Sarah Martindale, Sanya Tahmina Jhara, Mohammad Jahirul Karim, Muhammad Mujibur Rahman, Abdullah Al Kawsar, Abul Khair, ASM Sultan Mahmood, AKM Fazlur Rahman, Salim Mahmud Chowdhury, Susan Kim, Hannah Betts, Mark Taylor, Louise Kelly-Hope

**Affiliations:** 1 Centre for Neglected Tropical Diseases, Department of Tropical Disease Biology, Liverpool School of Tropical Medicine, Liverpool L3 5QA, UK; hayley.mableson@liverpool.ac.uk (H.M.); sarah.martindale@lstmed.ac.uk (S.M.); hannah.betts@lstmed.ac.uk (H.B.); mark.taypor@lstmed.ac.uk (M.T.); Louise.Kelly-Hope@lstmed.ac.uk (L.K.-H.); 2 Institute of Infection, Veterinary and Ecological Sciences, University of Liverpool, Liverpool L69 3BX, UK; 3 Filariasis Elimination and STH Control Program, Ministry of Health and Family Welfare, Dhaka 1000, Bangladesh; sanya.tahmina@gmail.com (S.T.J.); jahirulkarim@gmail.com (M.J.K.); drmujib.rahman@gmail.com (M.M.R.); dr.akawsar@gmail.com (A.A.K.); abulkhair018@gmail.com (A.K.); syeed25@gmail.com (A.S.M.); 4 Centre for Injury Prevention and Research Bangladesh, Dhaka 1206, Bangladesh; fazlur@ciprb.org (A.F.R.); smchow_dhaka@yahoo.com (S.M.C.); 5 College of Medicine and Public Health, Flinders University, Bedford Park SA 5042, Australia; sus97nz@gmail.com

**Keywords:** lymphedema, lymphatic filariasis, self-care, tissue tonometry, lower extremity, massage, breathing

## Abstract

Background: Lymphatic filariasis (LF) is a major cause of lymphedema, affecting over 16 million people globally. A daily, hygiene-centered self-care protocol is recommended and effective in reducing acute attacks caused by secondary infections. It may also reverse lymphedema status in early stages, but less so as lymphedema advances. Lymphatic stimulating activities such as self-massage and deep-breathing have proven beneficial for cancer-related lymphedema, but have not been tested in LF-settings. Therefore, an enhanced self-care protocol was trialed among people affected by moderate to severe LF-related lymphedema in northern Bangladesh. Methods: Cluster randomization was used to allocate participants to either standard- or enhanced-self-care groups. Lymphedema status was determined by lymphedema stage, mid-calf circumference, and mid-calf tissue compressibility. Results: There were 71 patients in each group and at 24 weeks, both groups had experienced significant improvement in lymphedema status and reduction in acute attacks. There was a significant and clinically relevant between-group difference in mid-calf tissue compressibility with the biggest change observed on legs affected by severe lymphedema in the enhanced self-care group (∆ 21.5%, −0.68 (−0.91, −0.45), *p* < 0.001). Conclusion: This study offers the first evidence for including lymphatic stimulating activities in recommended self-care for people affected by moderate and severe LF-related lymphedema.

## 1. Introduction

Lymphedema is a symptom of lymphatic failure and manifests as chronic connective tissue disease [1,2]. Fluid, cells, and proteins usually removed by lymphatic pumping accumulate in the tissue spaces between the skin and underlying muscle, and over time chronic inflammatory processes change the tissue composition and architecture [2]. Early fluid-rich stages are followed by fibrosis and fatty deposits in the middle stages, and advanced stages are marked by hyperkeratosis, papillomatosis, and other skin diseases [3]. Lymphatic filariasis (LF), a vector-borne disease endemic to 72 low and middle income countries (LMICs) including Bangladesh, is a major cause of lymphedema worldwide [4]. The World Health Organization (WHO) Global Programme to Eliminate LF (GPELF) aims to concurrently interrupt transmission using preventive chemotherapy via mass drug administration (MDA), and deliver morbidity management and disability prevention (MMDP) services to people with clinical manifestations [5]. The acute manifestations of LF are known as acute attacks, and involve debilitating episodes of pain, fever, and skin breakdown [4]. Chronic diseases are primarily hydrocele and lymphedema, and while hydrocele can be reversed with surgery in most cases [6], lymphedema has no surgical or pharmaceutical cure and must be managed for life through home-based self-care [7]. After 15 years of programmatic activities, Bangladesh has completed MDA and morbidity mapping, and estimates are that over 30,000 people are living with LF-related lymphedema, around 25% of whom are affected by lymphedema at an advanced stage [8,9,10].

Approaches to lymphedema management in LMICs must be implemented at a sustainable cost to the patient, family, and community. As acute attacks are the greatest cause of distress and disability, the WHO guidelines are centered around a daily, or twice daily, hygiene-centered regime of meticulous washing and drying of the affected body parts to reduce the risk of secondary infections. Adherence to the hygiene regime has been shown to reduce the frequency and duration of acute attacks [11,12]. However, two systematic reviews on lymphedema self-care found that while these standard recommendations may reverse some clinical signs in early or mild stage lymphedema, hygiene alone is less effective in reducing lymphedema status in more advanced disease [13,14]. Other than simple exercises and elevation, recommended activities do not address the underlying lymphatic failure, and although lymphatic massage and compression therapies are recommended for advanced lymphedema, the WHO aide-memoire for national program managers concedes that, *“The complete set of measures is more complex but usually cannot be implemented in resource-poor settings.“ [7]*.

Evidence-based self-care activities practiced by other populations, such as people who develop lymphedema after treatment for cancer, have not been trialed among people affected by lower-limb lymphedema in LMIC. While compression therapies are expensive and impractical in many LF-settings, activities shown to support lymphatic function such as deep breathing and self-massage [13] require few resources and should not increase the financial burden to the family or community. Drawing on evidence from research on cancer-related lymphedema, an enhanced self-care protocol was developed and a detailed description has been published [15].

Objective measurements of lymphedema status range from simple tape measures of limb circumference to nuclear imaging. No single measure is accepted as a universal standard and a variety of measures is generally required to determine lymphedema status [1]. Low-cost measures such as stage of lymphedema and limb circumference are commonly used in resource-poor settings to provide empirical evidence for the efficacy of lymphedema interventions, but in more advanced lymphedema these gross changes in limb status take a long time to reverse [16]. To support national scale up of effective MMDP programs in LMICs, affordable and field-friendly devices are needed to quantify early and clinically relevant improvements in lymphedema status, and several methods are available to detect the sub-clinical changes which precede overt improvements including ultrasonography, bio-impedance spectroscopy, and tissue tonometry [17,18,19]. Tissue tonometry has been used to quantify tissue compressibility or stiffness as a measure of change in lymphedema associated pathology since 1992 [20], and is frequently used in research on cancer-related lymphedema to measure changes in fibrotic induration and hyperkeratosis [20,21,22]. More recently it has been used to detect subclinical lymphatic associated change among asymptomatic young people in LF endemic regions of Papua New Guinea and Myanmar [19,20]. In the Myanmar cohort, a handheld tonometer, the Indurometer (Flinders and SA Biomedical Engineering, Australia), was able to detect a significant increase in mid-calf tissue compressibility among asymptomatic young people who were infected with LF, indicating that free fluid had begun to accumulate in the connective tissue spaces, even though there was no clinical sign of swelling. The Indurometer has demonstrated good intra-rater reliability in several populations including people affected by lower-limb lymphedema in this study [21,23,24,25].

The WHO guidelines on lymphedema management are interpreted and implemented by LF endemic countries according to national health policy, and in Bangladesh the national LF Elimination Program within the Ministry of Health and Family Welfare (MOHFW) delivers lymphedema services to registered patients via community clinics (CCs). Patients and their caregivers are taught the core components of self-care including washing and drying the affected body parts twice per day, attending to entry lesions and interdigital lesions, range of motion exercises, and limb elevation. A kit-box containing soap, washcloths, and medicated creams is provided, and patients can access further supplies such as medicated creams at the CC. To determine if an enhanced lymphedema self-care protocol incorporating activities for lymphatic stimulation can improve lymphedema status among people affected by moderate to severe lymphedema, a clinical trial which compared the enhanced self-care protocol to the WHO recommended standard self-care protocol was conducted. Objective measures of lymphedema status were collected including lymphedema stage, mid-calf circumference, and mid-calf tissue compressibility. The trial was conducted in Bangladesh and Ethiopia, which have contrasting environments and stages of GPELF activities, and the results on the Bangladesh cohort are presented here.

## 2. Materials and Methods

### 2.1. Study Design, Research Personnel, and Recruitment

**Figure 1:**
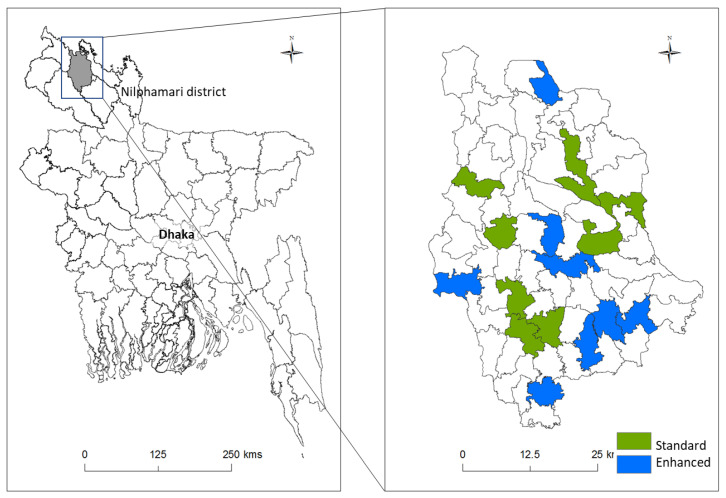
Map of all included community clinics in Nilphamari District, Bangladesh.

The prevalence of lymphedema is highest in the northern areas of Bangladesh [8], and Nilphamari District was chosen as it was not due to receive routine MMDP training during the study period. A random number generator was used to select 20 CCs from a list of all CCs in Nilphamari. Using cluster randomization, each CC was then allocated to either the standard self-care (control), or enhanced self-care (intervention) group and all participants at each CC were trained in the same self-care protocol. Follow-up measures and questionnaires were administered at four, twelve, and 24 weeks. Figure 1 is a map of all included CCs in the Nilphamari District by group.

Two research teams were formed by personnel from the MOHFW National LF Elimination Program and the Centre for Injury Prevention and Research, Bangladesh in Dhaka. Each research team comprised a clinical officer, data collector, research assistant, self-care trainer, and team supervisor.

At each CC, up to ten adults (≥18 years of age) affected by lymphedema at stage 3 or more according to Dreyer, et al. [26], and a designated adult caregiver, were invited to participate in the study. Two community health workers (CHW) at each CC were enrolled to be trained in the allocated protocol and to provide ongoing support. Each participant (patient, caregiver, and CHW) was provided with a written information sheet to keep in local language (Bangla), and the information was also explained by research staff in Bangla. Volunteers were pre-screened by the supervisor and trainer to ensure they had lymphedema of at least stage 3 in one or both legs [26], and where this was inconclusive, the patient was enrolled, and the clinical officer determined eligibility. Pre-screening also ensured that all patients had an adult caregiver available and that patients were excluded from the study if they had other known co-morbidities which may contribute to edema such as diabetes or congestive heart failure. The maximum of ten patients in each study center avoided over-loading the CHWs, but all volunteers who were not enrolled in the study were invited to participate in the lymphedema self-care training sessions.

### 2.2. Outcomes Measures and Data Collection

Data collection methods have been published previously [11,13]. Primary outcome measures were change in lymphedema status as determined by (1) lymphedema stage [26], (2) mid-calf limb circumference, and (3) mid-calf tissue compressibility. Secondary outcome measures were (1) frequency and (2) duration of acute attacks and (3) days of work lost due to lymphedema. To record adherence to the self-care protocol, a daily journal was kept by patients. Both legs of each patient were assessed and affected legs were classified as stage 1–7 according to clinical signs [26], and legs without any sign of lymphedema were classified as stage 0. Circumference and tissue compressibility measures were taken at the mid-point between the popliteal crease and the base of the heel of each leg with the patient lying prone. Circumference was measured with a retractable tape measure and the clinical officers were provided with a laminated instruction sheet (Supplementary Figure S1). All data were collected in the morning and not later than 2 pm to avoid variations in fluid accumulation that typically present towards the end of the day.

Tissue compressibility was quantified using the Indurometer (BME-1563; Flinders and SA Biomedical Engineering, Bedford Park, Australia) and the protocol was published in an intra-rater reliability analysis on the study population [23]. Briefly, the Indurometer is a handheld, battery-operated unit with a 1-centimeter (cm) diameter indenter projecting through a 7-cm diameter Perspex^®^ reference plate. The measure is taken with the indenter perpendicular to the measurement point and the reference plate parallel to the skin. The operator presses the device evenly into the skin and while the reference plate remains on the skin, the indenter progresses as far as is permitted by the underlying tissue. Once a load of 200 gm has been applied, a digital readout displays the distance the indenter was able to extend beyond the reference plate (millimeters). Lower scores indicate more tissue stiffness (less compressibility) and a higher score indicates softer tissue (more compressibility). The Indurometer has a self-calibrating procedure that was performed daily before the first measure and the clinical officers were provided with a laminated instruction sheet (Supplementary Figure S2).

Questionnaires were used to collect socio-demographic information and patients were asked about their medical status and lymphedema history. Data on acute attacks and days of work lost were elicited by recall over the previous one and six months [27]. The clinical officer administered the study questionnaires and collected the objective measures, and the data collector entered the answers and scores to the Open Data Kit Collect (ODK Collect) [28] application loaded to an electronic tablet (Samsung Galaxy Tab A 10.1). A paper journal for each 4-week period and a pencil were provided.

### 2.3. Intervention

The detailed protocol for both groups and a description of the material supplied to the patients has been published previously [15], a summary of which can be found in Supplementary Table S1. Patients, caregivers, and CHWs in both study groups were trained in the core components of standard lymphedema self-care including education on the cause of lymphedema. Patients were given the MOHFW National LF Elimination Program kit-box of materials, which was modified to include a pair of nail clippers, and could access support and additional medication if needed from the CC as per standard MOHFW policy and procedure. The intervention group was additionally trained in enhanced self-care components including deep breathing, leg exercises performed in the supine position, and lymphatic massage. A 500-mL bottle of coconut oil was provided as a medium to perform the massage, but people were able to use any other lubricant, or no lubricant, according to their preference. In addition, there were recommendations to walk at least 15 min twice per day, drink at least 5 glasses of fresh water daily, and to eat fresh fruit and vegetables on at least 4 days every week. A color-printed brochure with diagrams of the allocated self-care protocol was provided to each patient (Supplementary Figure S3). Patients and caregivers were trained in journal keeping and at each follow-up, patients were reminded of their self-care training and had their journals checked for adherence to the allocated activities.

The study was conducted in accordance with the Declaration of Helsinki [29] and approved by the Bangladesh Medical Research Committee and the Liverpool School of Tropical Medicine Research Ethics Committee (approval no. 012-18), and registered on the ISRCTN Registry, trial number 16764792. All subjects gave their informed consent for inclusion before they participated in the study. The study sponsor and funders had no role in study design or protocol development and no input into publication of study outcomes.

### 2.4. Analysis

Patient biometric measures were collected at baseline including height and weight, and leg dominance was determined by asking the question, “Which leg would you use to kick a ball?”. Lymphedema stages 1 and 2 were considered mild, stages 3 and 4 moderate, and stages 5–7 severe [17]. The stage of the most severe leg at baseline was used for whole person variables such as number of acute attacks, and the stage of each leg at baseline was included as a fixed effect for circumference and Indurometer scores. Circumference scores were derived from a single measure on each leg and the Indurometer score was the average of three successive measures. The Indurometer units were not available at baseline and therefore measures of tissue compressibility at the 4-week follow-up were considered as baseline for Indurometer data. 

The frequency and duration of acute attacks, and the number of lost working days due to lymphedema in the previous one month and six months, were analyzed using Poisson logistic mixed effects models. Time point, group (intervention or control), and maximum stage (none, moderate, or severe) at baseline were included in the model as fixed effects. Random effects were used for cluster and patient. After adjusting for stage at baseline, leg dominance was not a significant factor in this cohort, and Indurometer and circumference data were analyzed as right and left legs. In keeping with previous studies on lymphedema, a change of either ≥10% or ≥2cm in circumference score was considered clinically relevant and for Indurometer scores a change of ≥10% was used [30,31]. Adherence to the self-care protocol was assessed as performance of the allocated self-care regimen on at least 5 days per week.

All analyses were performed using Stata 15.1 (StataCorp, College Station, TX, USA) and a *p*-value of less than 0.05 was considered statistically significant. Descriptive statistics are presented as median with interquartile range for continuous variables and as frequency with percentage for categorical variables. Linear mixed effects models were used to analyze Indurometer and circumference measurements for each leg using the identity number as a random effect to account for non-independence of the leg pairs belonging to one individual. Time point and group (intervention or control) were included in the model as a priori. Leg dominancy and stage (none, mild, moderate or severe) at baseline were tested for inclusion in the model as fixed effects. All interactions were examined for inclusion. Random effects were used for cluster, patient, and leg (right or left) for Indurometer and circumference.

## 3. Results

### 3.1. Participants

**Figure 2:**
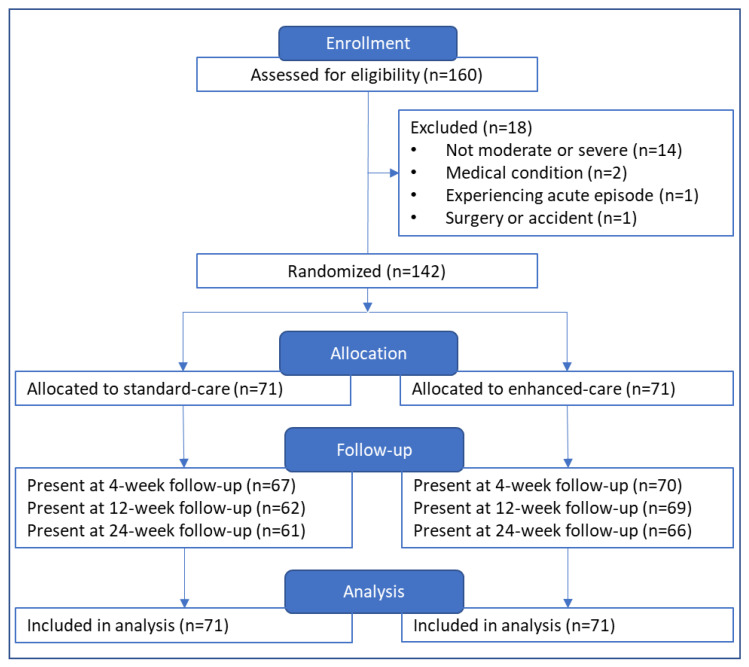
Flow chart of patients through the study.

A total of 160 patients, 160 designated caregivers and 36 CHWs were enrolled. Eighteen patients (and their corresponding caregivers) were excluded, mainly due to being assessed as not having at least one leg affected by stage 3 lymphedema or higher (n = 14). Of the 142 patients who were included in the study, 71 patients and their caregivers were allocated to the standard self-care (controls), and 71 to the enhanced self-care (intervention) group. Over 90% of patients attended the 4- and 12-week follow-ups and there was 89% return for the final 24-week measures (control 86%, intervention 93%, *p* > 0.05). A flow chart of all patients through the study can be seen in Figure 2.

There were no significant differences between groups at baseline for age, gender, stage of lymphedema, or circumference scores. There were also no differences in the number of acute attacks and working days lost due to lymphedema over either the previous one month, or six months (Table 1). At baseline, data were available on 284 legs and most were affected by moderate lymphedema (stages 3 and 4, n = 114 legs), 54 legs were affected by severe disease (stages 5–7), and 112 had no clinical sign of lymphedema (stage 0). Four legs were assessed as mild (control = 1 leg, intervention = 3 legs) and although these legs were included in the analysis model, there were too few to report on the results (Supplementary Table S2).

Adherence to the allocated self-care routine increased in both groups over the course of the study (control = 63–89%, intervention = 71–90%) and there were no between-group differences. At baseline, caregivers spent a cumulative median of 3 days per week supporting lymphedema care and this had increased to 4 days by 24 weeks (both groups).

### 3.2. Primary Outcomes

Change in lymphedema status was determined by a combination of limb stage, mid-calf circumference, and mid-calf tissue compressibility. The proportion of people affected by severe lymphedema fell from 19% at baseline to 9% at 24 weeks, as did the number of individual legs affected by severe lymphedema (control 22.5–14.8%, intervention 15.5–11.4%). The proportion of legs assessed as having no lymphedema also reduced slightly (control 38.0–35.2%, intervention 40.8–37.9%) while legs assessed as moderate increased (control 38.7–50.8%, intervention 41.5–49.2%). Over the study period, 16.1% of legs had reverted to a lower stage (controls n = 20, intervention n = 21), 6.3% progressed to a higher stage (controls n = 7, intervention n = 9), and three legs assessed as stage 0 at baseline had progressed to stage 3. Of the four legs affected by mild lymphedema at baseline, three legs progressed from stage 2 to 3, and the one leg at stage 1 had reverted to stage 0 at 24 weeks. None of the within-group changes were significant, and there were no significant between-group differences (Supplementary Table S2).

**Figure 3:**
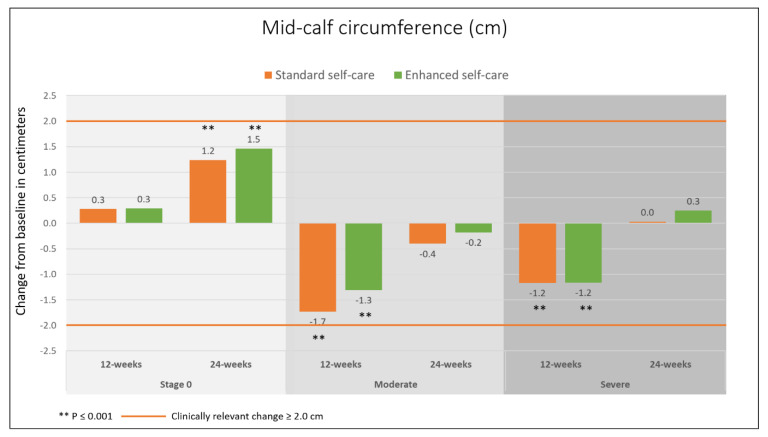
Change in mid-calf circumference from baseline by stage and group.

Mid-calf circumference measures at baseline ranged between 19.4 and 52.3 cm. Overall, legs affected by severe lymphedema in both groups were larger at mid-calf than legs without lymphedema by 12 (49.6%) and 11 cm (45.5%) on the left and right legs respectively (Supplementary Table S3). There were significant reductions in circumference measures on legs affected by moderate and severe lymphedema in both groups at the 4- and 12-week follow-ups, but none were clinically relevant (neither ≥10% or ≥2 cm). The biggest reduction was observed at 4 weeks on legs affected by moderate lymphedema (control −1.53 cm (4.8%, *p* < 0.001), intervention −1.21 cm (3.9%, *p* < 0.001), and for severe cases the biggest reduction was observed at 12 weeks (control −1.17 cm (3.3%, *p* = 0.001), intervention −1.16 cm (3.2%, *p* = 0.001) (≥ 2 cm, nor ≥ 10%). Legs that were unaffected by lymphedema at baseline (stage 0) also had a slight reduction in circumference at 4 weeks (control −0.42 cm (1.7%, *p* = 0.205), intervention −0.1 cm, (0.4%, *p* = 0.743), but at 24 weeks the mid-calf circumference was significantly larger than at baseline in both groups (controls +1.24 cm (5.1%), intervention +1.46 cm (6.0%), *p* < 0.001 for both). Change in circumference measures from baseline to 12 and 24 weeks can be seen in Figure 3.

Indurometer measures at the 4-week follow-up were considered as baseline data for mid-calf tissue compressibility. Scores ranged between 1.37 and 5.70 with legs affected by severe lymphedema returning significantly lower scores compared to legs without lymphedema (severe (3.13 ± 0.89), stage 0 (3.47 ± 0.70), *p* = 0.008). At baseline (4 weeks), there was a significant between-group difference in whole cohort Indurometer scores (adjusted difference of −0.31 (95% CI −0.55, −0.07), *p* = 0.012), but when legs were stratified by stage there were no between-group differences (*p* = 0.086) (Supplementary Table S4).

**Figure 4:**
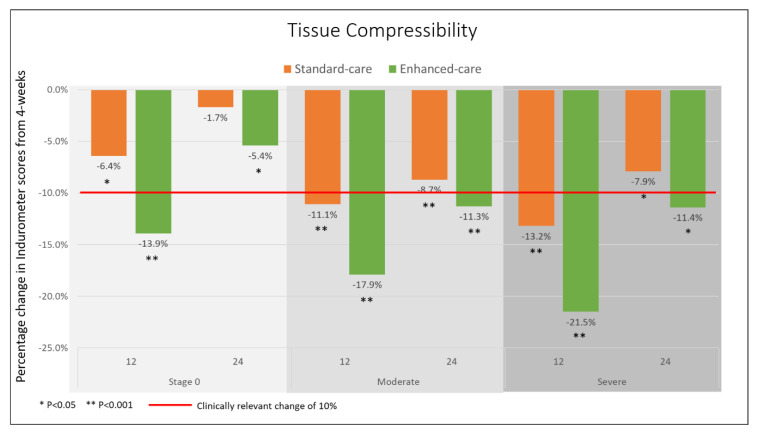
Change in Indurometer scores from baseline (4 weeks) by stage and group.

After accounting for the between-group differences at 4 weeks, there were significant between-group differences at the 12- and 24-week follow-ups (*p* < 0.001 for each time point, *p* = 0.016 for overall interaction between group and time). There were clinically relevant within-group changes (≥10%) in both groups with the largest change observed on legs affected by severe lymphedema in the intervention group at 12 weeks (21.5%, −0.68 (−0.91, −0.45), *p* < 0.001). At 24 weeks, the direction of the change had reversed and while neither group returned completely to baseline (4 weeks), only the intervention group retained a clinically relevant change compared to baseline (moderate cases 11.3% −0.41 (−0.57, −0.24), *p* < 0.001, severe cases 11.4%, −0.36 (−0.59, −0.12), *p* = 0.003). Change in Indurometer scores by stage and group at 12 and 24 weeks are shown in Figure 4.

It should be noted that lymphedema stage as an indicator of skin pathologies and tissue fibrosis, and limb circumference as an indicator of limb swelling, both have a linear relationship with lymphedema progression [1,31]. However, since tissue compressibility is determined by multiple tissue components, Indurometer scores may capture changes in skin pathologies, extracellular fluid loads and connective tissue fibrosis, and the direction of change should be interpreted in association with other available objective measures [1,30,31]. 

### 3.3. Secondary Outcomes

**Figure 5:**
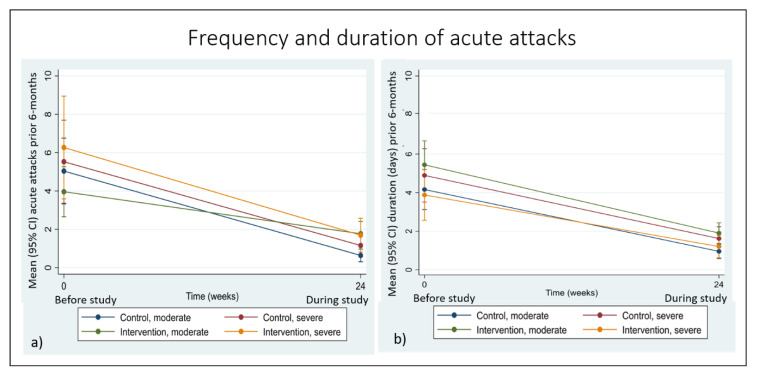
(**a**) Frequency and (**b**) duration of acute attacks by group and stage over 24 weeks.

At 24 weeks, both groups reported significant within-group reductions in the frequency and duration of acute attacks but there were no significant between-group differences. At baseline, more than 95% of patients (n = 136) reported that they had experienced at least one acute attack due to lymphedema and of these, more than 95% had experienced at least one episode in the six months prior to study commencement (n = 133, range 1–24 acute attacks, mean 4.77 ± 4.76, median 3 (IQR 2, 6)). Each episode had lasted an average of 4.67 ± 3.22 days and twelve patients reported episodes lasting 10 days or more (range 1–20 days, median 4 (IQR 3,5)). After 24 weeks, people affected by severe lymphedema reported the biggest decrease in frequency (controls −4.37 episodes, 1.16 (0.58, 1.73), intervention -4.55 episodes, 1.69 (0.81, 2.57)), while people affected by moderate lymphedema had experienced the best reduction in the duration of each episode (median -−4 days, (IQR −2, −5)) with no-one reporting episodes of 10-or-more-days duration (Figure 5).

Patient reports on the frequency and duration of acute attacks over the previous one month follow a similar pattern with more than 70% of patients reporting one or more acute attacks during the previous one month at baseline (n = 101, range 1–7 episodes, mean 4.46 ± 4.15, median 1 (IQR 0, 1)) and an average duration of 5.08 ± 3.56 days (median 4 (3, 5)). At 24 weeks, less than 30% of patients reported any acute episodes in the previous one month (n = 38, range 1–7, mean 0.53 ± 1.11, median 1 (IQR 0, 1)) and the mean duration of each episode was 3.82 ± 1.43 days (median 3.5 days (3, 4)). People affected by severe lymphedema reported the best reduction in frequency (controls −0.74 episodes, 0.44 (0.18, 0.70), intervention −0.16, 0.85 (0.42, 1.28)) and moderate cases experienced the best reduction in duration (controls, −3.57 days 0.90 (0.50, 1.31), intervention −1.79 days 1.20 (0.76, 1.63), Supplementary Table S5.

**Figure 6:**
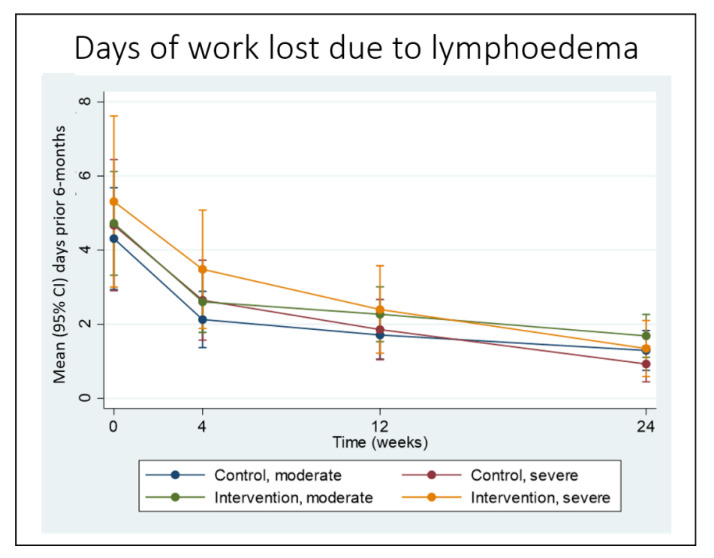
Workdays lost due to lymphedema by group and stage in the previous one month.

More than 90% of patients (n = 128) reported losing at least one day of work due to lymphedema in the six months prior to study commencement, but at 24 weeks only 40% of patients reported one-or-more workdays lost during the study period (*p* < 0.001). People affected by severe lymphedema were both the most impacted at baseline and reported the biggest improvement, but there were no significant between-group differences. People affected by severe lymphedema in the control group reported a median of 25 (IQR 13, 37) working days lost due to lymphedema in the six months prior to the study, but only 5 (3, 8) working days lost during the study period. In the intervention group, the median reduction among severe cases was from 26 (IQR 12, 39) days at baseline to 4 (2, 7) days at 24 weeks (Supplementary Table S6). Patient reports on workdays lost in the previous one month also fell at every follow-up in both groups (*p* < 0.001), Figure 6.

## 4. Discussion

Objective assessment of lymphedema status among people affected by lower-limb lymphedema in Northern Bangladesh found that as lymphedema stage increased, mid-calf circumference, and tissue stiffness also increased, which is consistent with known lymphedema progression [1,2,31]. After 24 weeks of performing either the standard WHO recommended self-care protocol (control), or an enhanced self-care protocol (intervention), predictable improvements in lymphedema status were also recorded including a small but significant reduction in limb circumference and some reversal of lymphedema stage, but these measures alone were unable to distinguish the between-group effects of the intervention. When tissue compressibility was considered, significant between-group differences and clinically relevant within-group changes were found, and these were greater among the intervention group who performed lymphatic stimulating activities such as self-massage and deep breathing as part of their daily lymphedema self-care.

Imperceptible changes in tissue composition precede clinical signs of lymphedema and a latent or stage 0 lymphedema may be present for many years before overt changes such as dermal sclerosis, fibrotic induration, and limb enlargement appear [13,17,21,27]. Such chronic changes also require long-term treatment to visibly reverse, and improvement generally begins with a reduction in excess free fluid as this component is the easiest to move. Fluctuations will be greater in the early fluid-rich stages of lymphedema than in the more advanced stages when fluid volume has reduced and become less “free” [31]. This is demonstrated by a larger change in circumference measures among moderate cases than among severe cases in both groups. Tissue compressibility also declined initially as the “cushion” of excess fluid was drained away, and at 12 weeks the significant between-group and within-group differences in Indurometer scores were large enough to be clinically relevant, with the greatest change recorded among severe cases performing the enhanced-care activities. While mid-calf circumference had essentially returned to baseline in limbs affected by lymphedema, tissue compressibility did not, indicating that other changes in tissue composition were occurring in addition to movement of free fluid. Although the study period was not long enough to detect significant changes in gross clinical signs, the overall improvement in stage at 24 weeks indicates that skin and tissue pathology had begun to improve, and suggests that the significant between-group differences in tissue compressibility at 24 weeks were due to presence of subclinical changes in tissue structure. Since these pre-cursor changes led to overtly detectable changes in lymphedema progression, the size and significance of the changes detected by Indurometry were an indication of the different rate of change between the study groups. 

In keeping with multiple other studies [13,14] we found meaningful improvements in the frequency and duration of acute attacks which is no doubt due to the hygiene-centered activities included in both protocols. In a comparison of plain and antibacterial soaps, Addis and colleagues [12] also reported the best improvement in acute attacks among the most severe cases and this was not related to the type of soap used, demonstrating that the hygiene-related nature of the protocol alone is enough to reduce acute episodes [27]. In our study, legs affected by moderate lymphedema had the most improvement at 4 and 12 weeks while severe cases tended to improve more slowly and consistently to 24 weeks. This is in keeping with the findings of Kerketta et al. [32] who noted that the efficacy of the footcare intervention varied with lymphedema grade. In that study, which compared standard self-care to standard self-care plus either of two drug regimens over 12 months, circumference reduction in all groups was greatest among legs at the earlier stages of lymphedema, while more advanced cases had the best improvement in the frequency of acute attacks. Likewise, legs in our study which had been assessed as stage 0 at baseline recorded the biggest fluctuations in limb circumference and were the only limbs to have a significant increase at 24 weeks. These studies illustrate that the opportunity to remove free fluid and reduce swelling declines as lymphedema stage advances, and support the implementation of lymphedema self-care at the earliest possible stage. 

People affected by severe lymphedema also suffer the most interruption in their ability to perform work, and in our study there were significant improvements in the number of working days lost due to lymphedema over the study period. This benefit may be amplified by the flow-on effect of freeing up caregivers who also lose workdays due to caring for people affected by lymphedema, and whose time is most frequently required during acute attacks [33,34]. As severe lymphedema improves more slowly than earlier stages, studies of longer duration are needed to determine whether enhanced self-care delivers better long-term benefits than standard self-care for these patients, but given the enormous economic benefits that can be returned to communities once lymphedema is managed [35,36,37], ascertaining the most effective self-care activities is worth investigating.

The risk of LF transmission in Bangladesh is very low [8], so new infections are unlikely to account for any change in lymphedema status. Likewise, adherence to the allocated protocol was high in both groups, and increased over the duration of the study, so non-performance of the self-care activities was also not a factor in the apparent “rebound” in some measures between 12 and 24 weeks. Nevertheless, the necessity for routine patient monitoring and support has been reported in several studies, and regular health worker support, patient, and caregiver re-training, and replenishment of supplies are needed to achieve long-term results [11,27,38,39]. The implementation guidelines for LF MMDP programs include monthly follow-up of patients for 3 months with an annual review thereafter [7]. Therefore, endemic countries should be supported to develop sustainable, long-term strategies for delivery of this minimum requirement to even the most remote communities affected by lymphedema.

The main strength of this study was the use of the Indurometer, which has the ability to quantify subtle changes in connective tissue composition, and its inclusion as a primary outcome measure enabled a deeper insight into early treatment effects than could have been shown by limb circumference or lymphedema stage alone. However, there are limitations in study design and data collection, which should be considered when interpreting the results. Since connective tissue pathology in severe lymphedema takes years to accumulate and is removed very slowly, the study period was not long enough to determine if the intervention can achieve more overt improvements over current recommendations. The mid-point of the calf was chosen as it is the optimal point for assessing lower-limb tissue compressibility [24], and collecting the circumference measure at the same point allowed for these measures to be interpreted together. However, a single circumference measure does not provide much information about overall limb volume and the addition of another circumference measure at the ankle, the most typical area for swelling to accumulate, or the addition of other measures such as a portable 3D scanner to calculate limb volume may have provided a more robust measure of how the self-care protocols affected overall leg swelling. Whole cohort Indurometer scores at 4 weeks were significantly different between groups, but without pre-study data there is no way of knowing if this was as a result of differences achieved through the intervention, or if the between-group scores had already been different at baseline. Finally, although one of the criteria in choosing the enhanced self-care activities was to avoid additional financial burden to the family or community, no economic analysis was performed, and it is not known if the cost of materials or the cost of extra caregiver time had any significant economic impact. 

This is the first randomized trial to investigate the use of simple lymphatic-stimulating self-care activities, proven to be effective for cancer-related lymphedema by people affected by LF-related lymphedema. Although compression therapies have been shown to improve patient outcomes in LF endemic settings [40,41], they are expensive, impractical in most resource-poor rural settings, and may increase the risk of secondary infection [27]. Cost free, lymphatic stimulating activities such as deep abdominal breathing and self-massage are effective in improving lymphedema status, [13,42,43,44], but are rarely included in management of LF-related lymphedema [45,46]. Furthermore, the inability to objectively and easily measure early improvement in lymphedema status, especially for more advanced cases, has potentially led to underreporting on the effectiveness of lymphedema self-care programs. This has ramifications for providing evidence-based guidelines to LF endemic countries programs, and hampers efforts to increase private donor support for national MMDP program requirements. 

The Indurometer is a reliable, non-invasive, and field-friendly device which can detect and quantify covert changes in tissue composition before more overt changes can be seen. This, or other portable devices capable of capturing early indicators of change in lymphedema status should be included in implementation research on interventions for LF-related lymphedema. 

## 5. Conclusions

Most LF-endemic countries must work within limited budgets to deliver national-scale services to people affected by LF-related lymphedema, and therefore depend on expert guidance from WHO regarding the most effective, evidence-based treatment protocols. The significant changes in tissue composition detected by Indurometry in our study provide the first indication that inclusion of lymphatic-stimulating activities in lymphedema self-care protocols may benefit people affected by moderate to severe lymphedema. Factors associated with the loss of some benefit at 24 weeks, and the effects of lymphatic stimulation on early stage lymphedema require further investigation.

## Figures and Tables

**Table 1:** Participant characteristics at baseline.

		Standard Self-Care	Enhanced Self-Care	
		(Controls)	(Intervention)	*p* =
Participants		n = 71	n = 71	
Age, median (IQR)		55 (48, 65)	52 (45, 60)	0.16
Gender = female		55 (77%)	52 (73%)	0.56
Marital status = married		50 (71%)	59 (82%)	0.17
Highest education = illiterate		35 (50%)	48 (67%)	0.22
Maximum stage of lymphedema				0.22
= moderate	n (%)	43 (61%)	50 (70%)	
= severe	n (%)	18 (39%)	21 (30%)	
Acute attacks—last 1 month	Median (IQR)	1 (0, 1)	1 (0, 1)	0.86
Workdays lost—last 1 month	Median (IQR)	3 (0, 6)	4 (0, 7)	0.48
Acute attacks—last 6 months	Median (IQR)	3.5 (1.5, 6)	3 (2, 6)	0.74
Workdays lost—last 6 months	Median (IQR)	18 (8, 30)	18 (7, 30)	0.96
Circumference—left leg (cm)	Median (IQR) Mean (SD	29.2 (24.6, 33.8) 29.4 (5.91)	27.8 (24.2, 32.4) 28.6 (6.80)	0.48 0.41
Circumference—right leg (cm)	Median (IQR) Mean (SD)	30.5 (25.8, 34.1) 29.9 (5.75)	27.9 (24.9, 32.4) 29.2 (5.77)	0.25 0.40
Indurometer score—left leg *	Median (IQR) Mean (SD)	3.20 (2.71, 3.62) 3.17 (0.78)	3.45 (2.95, 4.02) 3.48 (0.82)	0.03 0.03
Indurometer score—right leg*	Median (IQR) Mean (SD)	3.51 (3, 3.86) 3.44 (0.76)	3.53 (3.18, 3.94) 3.56 (0.64)	0.40 0.34

Moderate lymphedema = stages 3–4, severe = stage 5–7. IQR = inter-quartile range, SD = standard deviation, * Indurometer measures at 4 weeks.

## Supplementary Materials

The following are available online at https://www.mdpi.com/2077-0383/9/8/2444/s1, Figure S1: Circumference measure instruction sheet, Figure S2: The Indurometer, Figure S3: Intervention protocols, Table S1: Summary of the self-care protocols, Table S2: Lymphoedema stage, Table S3: Mid-calf Circumference, Table S4: Indurometer scores, Table S5: Acute attacks, Table S6: Working days lost to lymphoedema.
