# Supplementary material for: Effect of an Enhanced Self-Care Protocol on Lymphedema Status among People Affected by Moderate to Severe Lower-Limb Lymphedema in Bangladesh, a Cluster Randomized Controlled Trial
Source: J Clin Med. 2020 Jul 30;9(8):2444. doi: 10.3390/jcm9082444 (PMC7464742; doi:10.3390/jcm9082444)

Supplementary Materials

Supplementary Figure 1. Circumference measure instruction sheet.

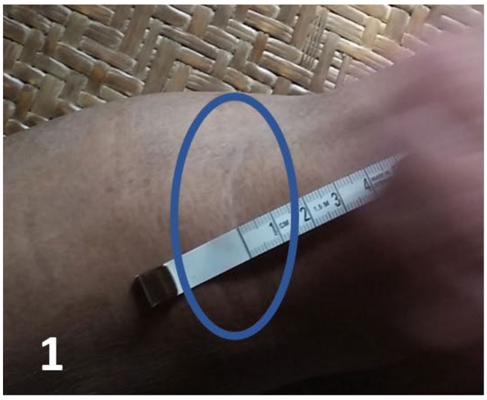

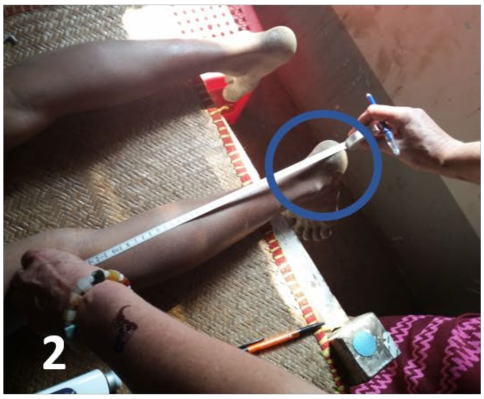

1. Place the ‘0’ mark of the tape measure at the **most obvious crease behind the knee**
2. Note the length at the **base of the heel** (one decimal place) on the ODK
3. The ODK will then give you the length of the mid-point
4. Hold the tape flat the skin with one finger on **either side of the mid-point**

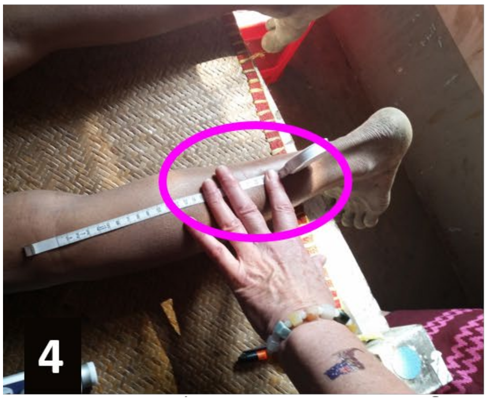

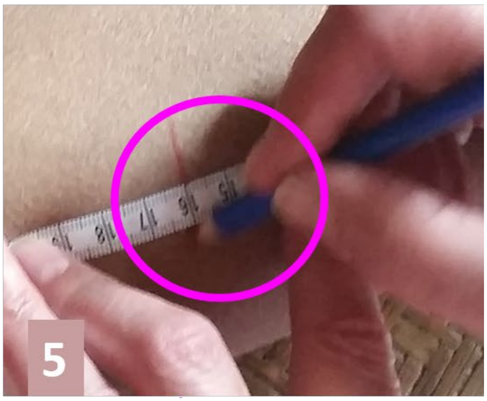

5. Using the skin marker, make a small horizontal line **exactly on both sides** of the nominated mid-point
6. Pass the tape under the leg and wrap the calf with the tape
7. Cross the tape so that **‘0’ end is above the pair of lines and the reel end is below**
8. Make sure that the **‘0’ is located between the two lines**
9. Read the **value on the tape which lines up with the ‘0’** and enter it to the ODK (one decimal place)

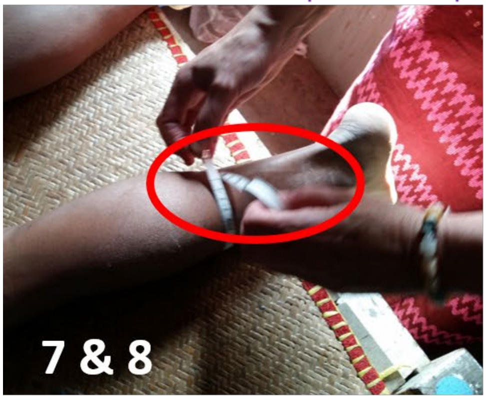

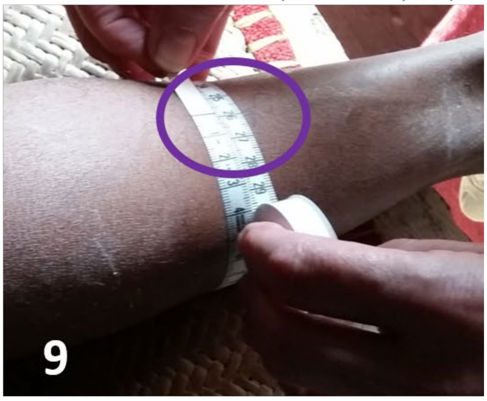

Supplement: Supplementary file 1 [file jcm-09-02444-s001.zip › SUPPLEMENTARY Figure 1.pdf]
