# Supplementary material for: Effect of an Enhanced Self-Care Protocol on Lymphedema Status among People Affected by Moderate to Severe Lower-Limb Lymphedema in Bangladesh, a Cluster Randomized Controlled Trial
Source: J Clin Med. 2020 Jul 30;9(8):2444. doi: 10.3390/jcm9082444 (PMC7464742; doi:10.3390/jcm9082444)
Supplement: Supplementary file 1 [file jcm-09-02444-s001.zip › SUPPLEMENTARY Figure 2.pdf]

Supplementary Figure 2. The Indurometer

Flinders and SA Biomedical Engineering, Australia

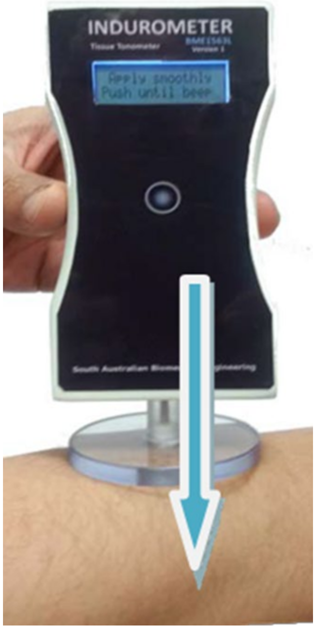

Indurometer calibration and instruction sheet

Calibrate each device once per day (before the first measure)
See over for calibration instructions

Use device 'A' for every measure.
If the device is not working use device 'B' and complete a protocol deviation form

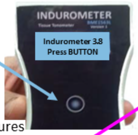

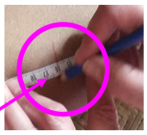

1. Push the button to begin the measure
2. The display will show-

   Apply smoothly
   Push until beep

3. Use the same midpoint mark on the calf that you used for the circumference measures
4. Align the plunger of the Indurometer with this mark
5. Keep the footplate parallel with the skin and press the device smoothly and gently into the skin
6. DO NOT HOLD it there. Remove it as soon as you hear the beep
7. If you hear a single beep the measure was successful and the score will be displayed with 2 decimal places-

   X.XX IU
   Press BUTTON

8. Record this score in your data collection form.
9. If you hear two beeps the measure was not successful. The screen will tell you if it was **too fast** or **too slow**
10. Reset the device and try again

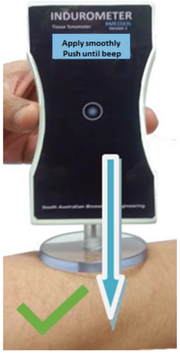

GOOD TECHNIQUE
The footplate is FLAT to the skin

DO NOT apply heavy pressure
DO NOT keep pressing after the beep

BAD TECHNIQUE
The footplate is NOT FLAT to the skin

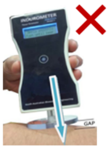

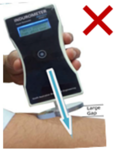

If the calf is narrower than the footplate

Align the footplate to the middle of the leg so that the **GAPS ON EITHER SIDE ARE EVEN** and the footplate is **AS FLAT AS POSSIBLE** along the length of the calf.

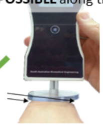

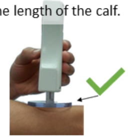

Calibrate both devices once per day (before the first measure)

Use device 'A' for every measure
If device 'A' cannot be calibrated, use device 'B' and complete a protocol deviation form

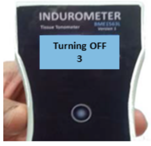

1. Turn the Indurometer OFF by pressing and holding the button for 5 seconds or until you see **Turning OFF 3** displayed on the screen. Wait until the screen is blank.
2. Press and hold the button for 5 seconds again until you see the display-

   CAL MODE
   Press BUTTON

3. Press the button again briefly and the screen will display-

   ZERO and CAL CHECK and then- **Hold Steady Press BUTTON**

4. Make sure nothing is touching the footplate or plunger and press the button again. The screen will display- **Zeroing Please wait**

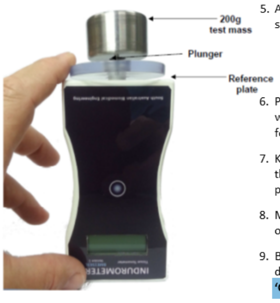

5. After a few seconds you will hear a beep and the screen with display-

   Invert. Add 200g
   Press BUTTON

6. Place the Indurometer upside down on a flat surface with the footplate at the top and gently push the footplate down half way.
7. Keeping the footplate held down, add the weight to the plunger - there is a hole in the weight where the plunger fits.
8. Make sure the weight is not touching the footplate or your finger or anything other than the plunger.
9. Briefly press the button and continue holding the device in this position. You will momentarily see **'Calibrating please wait'** on the screen.
10. After you hear a beep you can gently remove the weight and hold the Indurometer upright. The display will show some numbers like *123 456 789*. You can ignore this.
11. Briefly press the button again. There are two possible displays-

    CAL complete Press BUTTON or- **CAL required Press BUTTON**

If the calibration has been successful press the button again and you can begin the first measure

If the calibration was not successful, press the button and begin the calibration process again

DO NOT use alcohol based solvents to clean the Indurometer
