# Supplementary material for: Effect of an Enhanced Self-Care Protocol on Lymphedema Status among People Affected by Moderate to Severe Lower-Limb Lymphedema in Bangladesh, a Cluster Randomized Controlled Trial
Source: J Clin Med. 2020 Jul 30;9(8):2444. doi: 10.3390/jcm9082444 (PMC7464742; doi:10.3390/jcm9082444)

Supplementary Figure 3. Intervention protocols

Participant brochure on the allocates self-care activities – Standard self-care group

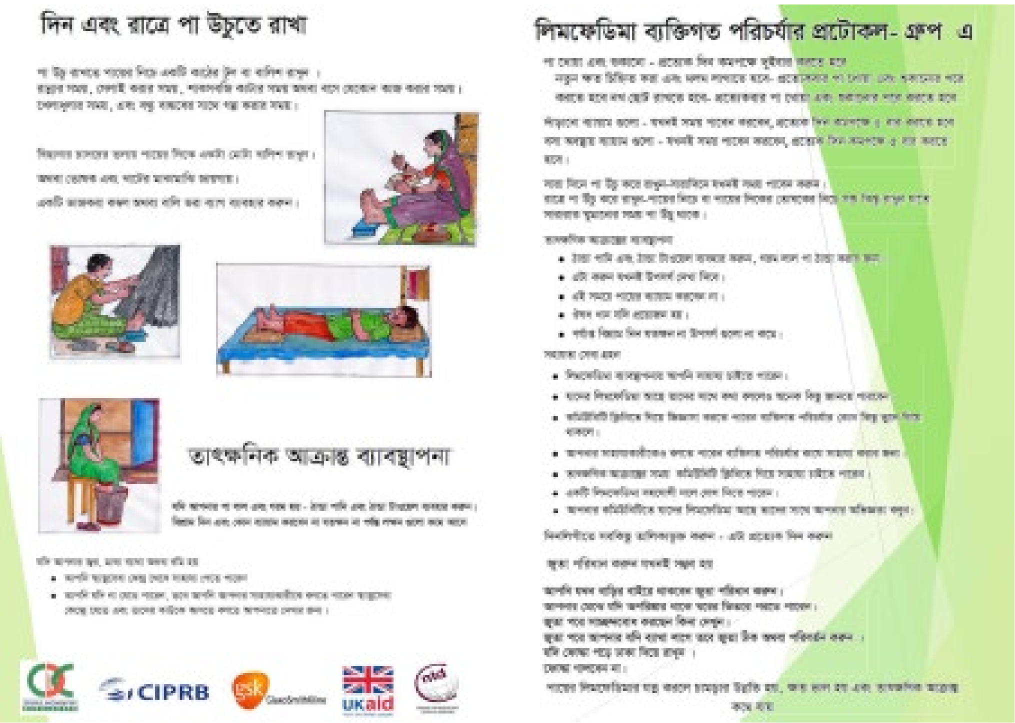

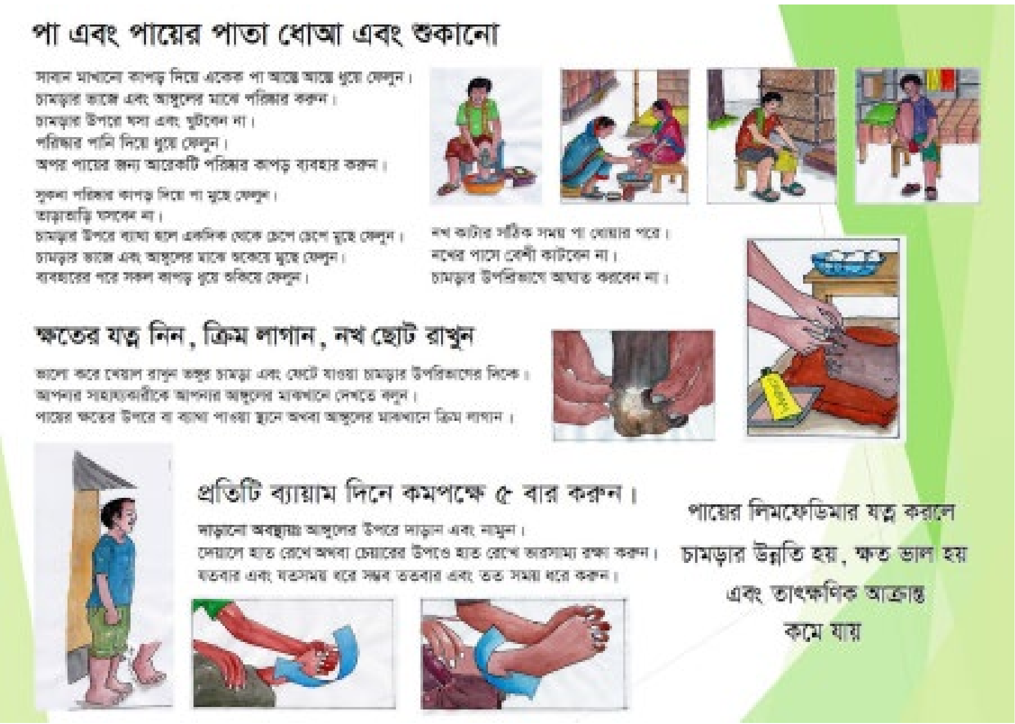

Participant brochure on the allocates self-care activities – Enhanced self-care group

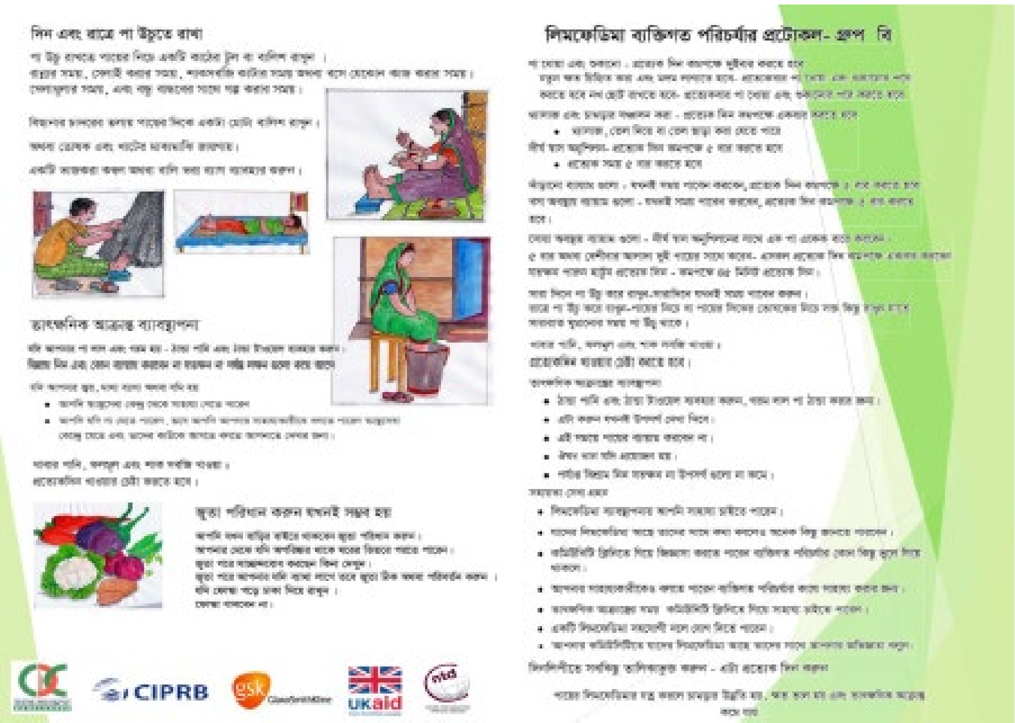

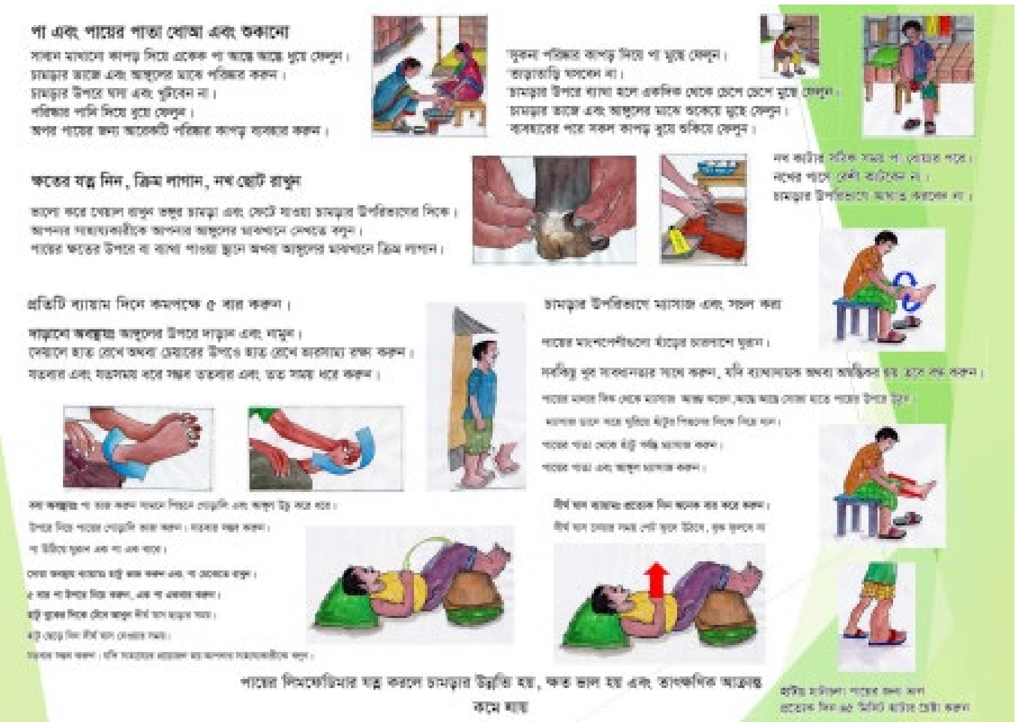

Supplement: Supplementary file 1 [file jcm-09-02444-s001.zip › SUPPLEMENTARY Figure 3.pdf]
