# Supplementary material for: Effect of an Enhanced Self-Care Protocol on Lymphedema Status among People Affected by Moderate to Severe Lower-Limb Lymphedema in Bangladesh, a Cluster Randomized Controlled Trial
Source: J Clin Med. 2020 Jul 30;9(8):2444. doi: 10.3390/jcm9082444 (PMC7464742; doi:10.3390/jcm9082444)
Supplement: Supplementary file 1 [file jcm-09-02444-s001.zip › SUPPLEMENTARY Table 1.pdf]

Supplementary Table 1. Summary of the self-care protocols

Summary of the standard self-care (control group) and enhanced self-care (intervention group) protocols

Activity	Group	Frequency	Materials supplied (timepoint)
Daily washing and drying of affected body parts	both	At least twice per day	2 bars of plain soap, 2 wash cloths & drying towels (B0,F12)
Treating entry lesions	both	Daily	1 tube of antibiotic cream & a gauze bandage (B0,F12)
Treating interdigital lesions	both	Daily	One tube of antifungal cream (B0,F12)
Trimming nails	both	As needed	Nail clippers (B0,F12)
Standing exercises	both	Once or twice per day	None needed
Seated exercises	both	Once or twice per day	None needed
Daytime elevation	both	Whenever possible	None needed
Overnight elevation	both	Every night	None needed
Massage	IG	Once or twice per day	500m l coconut oil (B0,F12)
Skin and tissue mobilisation	IG	Once or twice per day	None needed
Breathing exercises	IG	5 breaths at least 5 times per day	None needed
Lying down exercises	IG	5 movements each leg once or twice per day	None needed
Walking	IG	At least 15 minutes, 4 times per day	None needed
Water	IG	At least 5 glasses per day	Usual source
Fresh fruit and veg	IG	One serve on at least 4 days per week	Usual source

IG = Intervention Group B0 = baseline, F12= 12 week follow-up
