# Supplementary material for: Effect of an Enhanced Self-Care Protocol on Lymphedema Status among People Affected by Moderate to Severe Lower-Limb Lymphedema in Bangladesh, a Cluster Randomized Controlled Trial
Source: J Clin Med. 2020 Jul 30;9(8):2444. doi: 10.3390/jcm9082444 (PMC7464742; doi:10.3390/jcm9082444)
Supplement: Supplementary file 1 [file jcm-09-02444-s001.zip › SUPPLEMENTARY Table 2.pdf]

Supplementary Table 2. Lymphoedema stage

Proportion of legs by stage of lymphoedema and group at baseline and 24-weeks.

**Control Group**					
		Stage 0	*Mild*	*Moderate*	*Severe*
	Leg	*n (%)*	*n (%)*	*n (%)*	*n (%)*
Baseline					
n=142 legs	Left	29 (40.8)	1 (1.4)	30 (42.2)	11 (15.5)
	Right	25 (35.2)	0 (0.0)	25 (35.2)	21 (29.6)
	Total	**54 (38.0)**	**1 (0.7)**	**55 (38.7)**	**32 (22.5)**
24-weeks					
n=61	Left	22 (36.1)	0 (0.0)	32 (52.5)	7 (11.5)
	Right	21 (34.4)	0 (0.0)	30 (49.2)	10 (16.4)
	Total	**43 (35.2)**	**0 (0.0)**	62 **(50.8)**	**18 (14.8)**
	△ *B-24*	-3%	-0.7%	+ 12.1%	-8.4%
**Intervention Group**					
		Stage 0	*Mild*	*Moderate*	*Severe*
	Leg	*n (%)*	*n (%)*	*n (%)*	*n (%)*
Baseline					
n=71	Left	26 (36.6)	2 (2.8)	33 (46.4)	10 (14.1)
	Right	32 (45.1)	1 (1.4)	26 (36.8)	12 (16.9)
	Total	**58 (40.8)**	**3 (2.1)**	**59 (41.5)**	**22 (15.5)**
24-weeks					
n=66	Left	26 (39.4)	0 (0.0)	31 (47.0)	7 (10.6)
	Right	24 (36.4)	0 (0.0)	34 (51.5)	8 (12.1)
	Total	**50 (37.9)**	**0 (0.0)**	**65 (49.2)**	**15 (11.4)**
	△ *B-24*	-3.1%	-2.1%	+ 7.7%	-4.1%

△ *B-24 change from baseline to 24-weeks*
