# Supplementary material for: Effect of an Enhanced Self-Care Protocol on Lymphedema Status among People Affected by Moderate to Severe Lower-Limb Lymphedema in Bangladesh, a Cluster Randomized Controlled Trial
Source: J Clin Med. 2020 Jul 30;9(8):2444. doi: 10.3390/jcm9082444 (PMC7464742; doi:10.3390/jcm9082444)
Supplement: Supplementary file 1 [file jcm-09-02444-s001.zip › SUPPLEMENTARY Table 3.pdf]

Supplementary Table 3. Mid-calf Circumference

Baseline mid-calf circumference in cm by group and stage

Stage	Group	N (legs)	Mean	SD
Stage 0	Standard self-care	54	24.2	2.98
	Enhanced self-care	58	24.3	4.21
Moderate	Standard self-care	55	31.8	3.82
	Enhanced self-care	59	30.7	4.59
Severe	Standard self-care	32	35.4	4.49
	Enhanced self-care	22	36.6	5.21

SD = standard deviation

Change in mid-calf circumference from baseline to follow-up at 4-, 12- and 24-weeks by stage and group.

	4 weeks		12 weeks		24 weeks	
	Control	Intervention	Control	Intervention	Control	Intervention
Stage 0	-1.7%<br>-0.42 (-1.07, 0.23)<br>P=0.205	-0.4%<br>-0.1 (-0.72, 0.51)<br>P=0.743	+1.2%<br>0.28 (-0.39, 0.95)<br>P=0.409	+1.2%<br>0.29 (-0.33, 0.92)<br>P=0.356	+5.1%<br>1.24 (0.57, 1.91)<br>P<0.001	+6.0%<br>1.46 (0.84, 2.09)<br>P<0.001
Moderate	-4.8%<br>-1.53 (-2.07, -0.99)<br>P<0.001	-3.9%<br>-1.21 (-1.72, -0.7)<br>P<0.001	-4.2%<br>-1.33 (-1.87, -0.78)<br>P<0.001	-4.3%<br>-1.31 (-1.83, -0.8)<br>P<0.001	-1.3%<br>-0.40 (-0.95, 0.15)<br>P=0.156	-0.6%<br>-0.18 (-0.69, 0.33)<br>P=0.488
Severe	-3.6%<br>-1.27 (-1.92, -0.62)<br>P<0.001	-2.6%<br>-0.95 (-1.63, -0.27)<br>P=0.006	-3.3%<br>-1.17 (-1.83, -0.5)<br>P=0.001	-3.2%<br>-1.16 (1.84, -0.47)<br>P=0.001	+0.0%<br>0.03 (-0.63, 0.69)<br>P=0.931	+0.7%<br>0.25 (-0.44, 0.93)<br>P=0.477

Clinically relevant change ≥ 2cm
