# Supplementary material for: Effect of an Enhanced Self-Care Protocol on Lymphedema Status among People Affected by Moderate to Severe Lower-Limb Lymphedema in Bangladesh, a Cluster Randomized Controlled Trial
Source: J Clin Med. 2020 Jul 30;9(8):2444. doi: 10.3390/jcm9082444 (PMC7464742; doi:10.3390/jcm9082444)
Supplement: Supplementary file 1 [file jcm-09-02444-s001.zip › SUPPLEMENTARY Table 4.pdf]

Supplementary Table 4. Indurometer scores

Baseline Indurometer scores by stage and group.*

Stage	Group	N (legs)	Mean	SD
Stage 0	Standard self-care	52	3.44	0.78
	Enhanced self-care	58	3.53	0.63
Moderate	Standard self-care	53	3.33	0.67
	Enhanced self-care	59	3.64	0.74
Severe	Standard self-care	32	3.04	0.91
	Enhanced self-care	22	3.17	0.92

Change in Indurometer scores from week 4 to 12- and 24- weeks by stage and group.

	12 weeks		24 weeks	
	**Control**	**Intervention**	**Control**	**Intervention**
**Stage 0**	6.4%<br>-0.22 (-0.39, -0.04)<br>P=0.015	13.9%<br>-0.49 (-0.66, -0.33)<br>P<0.001	1.7%<br>-0.06 (-0.24, 0.11)<br>P=0.478	5.4%<br>-0.19 (-0.36, -0.02)<br>P=0.030
**Moderate**	11.1%<br>-0.37 (-0.54, -0.2)<br>P<0.001	17.9%<br>-0.65 (-0.81, -0.48)<br>P<0.001	8.7%<br>-0.29 (-0.46, -0.11)<br>P=0.001	11.3%<br>-0.41 (-0.57, -0.24)<br>P<0.001
**Severe**	13.2%<br>-0.4 (-0.62, -0.18)<br>P<0.001	21.5%<br>-0.68 (-0.91, -0.45)<br>P<0.001	7.9%<br>-0.24 (-0.45, -0.02)<br>P=0.035	11.4%<br>-0.36 (-0.59, -0.12)<br>P=0.003

Control = standard self-care, Intervention = enhanced self-care

Clinically relevant change ≥ 10%
