# Supplementary material for: Effect of an Enhanced Self-Care Protocol on Lymphedema Status among People Affected by Moderate to Severe Lower-Limb Lymphedema in Bangladesh, a Cluster Randomized Controlled Trial
Source: J Clin Med. 2020 Jul 30;9(8):2444. doi: 10.3390/jcm9082444 (PMC7464742; doi:10.3390/jcm9082444)
Supplement: Supplementary file 1 [file jcm-09-02444-s001.zip › SUPPLEMENTARY Table 5.pdf]

Supplementary Table 5. Acute attacks

Frequency and duration of acute attacks by group and stage previous 6 months.

	**Moderate**		**Severe**	
	**Control**	**Intervention**	**Control**	**Intervention**
**Frequency of acute attacks - Median (IQR)**				
**Baseline**	5.04 (3.32, 6.76)	3.96 (2.65, 5.27)	5.53 (3.36, 7.69)	6.27 (3.59, 8.95)
**24 weeks**	0.64 (0.31, 0.97)	1.77 (1.12, 2.41)	1.16 (0.58, 1.73)	1.69 (0.81, 2.57)
**Change in frequency**	-4.4	-2.19	-4.37	-4.55
**Duration in days - Median (IQR)**				
**Baseline**	4.16 (3.12, 5.20)	5.44 (4.20, 6.68)	4.89 (3.51, 6.28)	3.88 (2.57, 5.19)
**24 weeks**	0.95 (0.58, 1.33)	1.9 (1.37, 2.43)	1.62 (1.01, 2.22)	1.2 (0.63, 1.78)
**Change in days**	-3.21	-3.54	-3.27	-2.68

Control = standard self-care, Intervention = enhanced self-care

Frequency and duration of acute attacks by group and stage previous one month

	Moderate		Severe	
	Control	Intervention	Control	Intervention
Frequency of acute attacks - Median (IQR)				
Baseline *n=101*	0.89 (0.59, 1.19)	0.91 (0.63, 1.2)	1.18 (0.73, 1.62)	1.01 (0.54, 1.47)
4 weeks *n=84*	0.71 (0.38, 1.04)	0.62 (0.39, 0.85)	0.71 (0.38, 1.04)	0.91 (0.47, 1.35)
12 weeks n*=73*	0.59 (0.33, 0.84)	0.71 (0.46, 0.96)	0.62 (0.31, 0.94)	0.75 (0.35, 1.15)
24 weeks *n=34*	0.34 (0.14, 0.53)	0.59 (0.36, 0.81)	0.44 (0.18, 0.70)	0.85 (0.42, 1.28)
△ *B-24*	-0.55	-0.32	-0.74	-0.16
Duration in days - Median (IQR)				
Baseline	4.47 (3.10, 5.85)	2.99 (2.08, 3.91)	3.74 (2.33, 5.16)	4.15 (2.39, 5.91)
4 weeks	1.71 (1.09, 2.33)	2.58 (1.78, 3.39)	1.97 (1.14, 2.79)	2.90 (1.59, 4.21)
12 weeks	1.62 (1.01, 2.23)	1.59 (1.05, 2.13)	1.80 (1.02, 2.58)	1.87 (0.94, 2.81)
24 weeks	0.90 (0.50, 1.31)	1.20 (0.76, 1.63)	0.96 (0.47, 1.44)	1.35 (0.61, 2.08)
△ *B-24*	-3.57	-1.79	-2.78	-2.80

Control = standard self-care, Intervention = enhanced self-care
