# Supplementary material for: Effect of an Enhanced Self-Care Protocol on Lymphedema Status among People Affected by Moderate to Severe Lower-Limb Lymphedema in Bangladesh, a Cluster Randomized Controlled Trial
Source: J Clin Med. 2020 Jul 30;9(8):2444. doi: 10.3390/jcm9082444 (PMC7464742; doi:10.3390/jcm9082444)
Supplement: Supplementary file 1 [file jcm-09-02444-s001.zip › SUPPLEMENTARY Table 6.pdf]

Supplementary Table 6. Working days lost to lymphoedema

Working days lost due to lymphoedema at 4-, 12- and 24-weeks by stage and group

	Moderate		Severe	
	**Control**	**Intervention**	**Control**	**Intervention**
Number of days lost over the previous 6-months - Median (IQR)				
**Baseline**	21.25 (12.82, 29.68)	23.49 (14.53, 32.45)	25.06 (13.37, 36.74)	25.64 (12.41, 38.87)
**24-weeks**	2.30 (1.26, 3.33)	5.60 (3.38, 7.83)	5.46 (2.79, 8.14)	4.36 (1.88, 6.83)
	-18.95	-17.89	-19.6	-21.28
Number of days lost over the previous 1-month - Median (IQR)				
**Baseline**	4.31 (2.94, 5.68)	4.72 (3.32, 6.12)	4.67 (2.9, 6.44)	5.31 (3.00, 7.62)
**4-weeks**	2.13 (1.37, 2.89)	2.61 (1.78, 3.43)	2.65 (1.57, 3.73)	3.48 (1.88, 5.08)
**12-weeks**	1.71 (1.06, 2.36)	2.27 (1.53, 3.01)	1.86 (1.05, 2.67)	2.4 (1.22, 3.58)
**24-weeks**	1.29 (0.76, 1.83)	1.69 (1.11, 2.27)	0.93 (0.45, 1.41)	1.35 (0.59, 2.1)
	-3.02	-3.03	-3.74	-3.96

Control = standard self-care, Intervention = enhanced self-care
